# Reducing the barrier effect of graphene sheets on a Ag cocatalyst to further improve the photocatalytic performance of TiO_2_†

**DOI:** 10.1039/c8ra02268b

**Published:** 2018-04-16

**Authors:** Juanjuan Ma, Chaocun Zhou, Jinlin Long, Zhengxin Ding, Rusheng Yuan, Chao Xu

**Affiliations:** State Key Laboratory of Photocatalysis on Energy and Environment, College of Chemistry, Fuzhou University Fuzhou 350002 P. R. China cxu@fzu.edu.cn

## Abstract

Graphene-based cocatalysts can improve the photocatalytic properties of semiconductors, but sometimes, they also function as barrier-like materials, influencing the photoactivity of composites. However, in a multi-cocatalyst system, less attention is paid to these negative effects of graphene on the performance of other cocatalysts. In this study, by adjusting the loading sequence of graphene and Ag cocatalyst on the surface of TiO_2_ spheres, the barrier effect of graphene sheets on Ag nanoparticles could be controlled effectively. As a result, these ternary composites with almost no Ag nanoparticles wrapped by graphene possessed improved properties for the photocatalytic reduction of nitro-aromatics as compared to those with some Ag nanoparticles covered by graphene. Furthermore, this phenomenon of barrier effect caused by graphene could be found in the control reaction with metal silver as the main catalyst; this indicated that by avoiding the possible negative influence of graphene on other cocatalysts, the properties of composites with graphene-containing multi cocatalysts could be further improved.

## Abstract 2

By avoiding the possible barrier influence of graphene on other cocatalysts, the photocatalytic properties of the composites containing multi-cocatalysts could be further improved.

## Graphical abstract

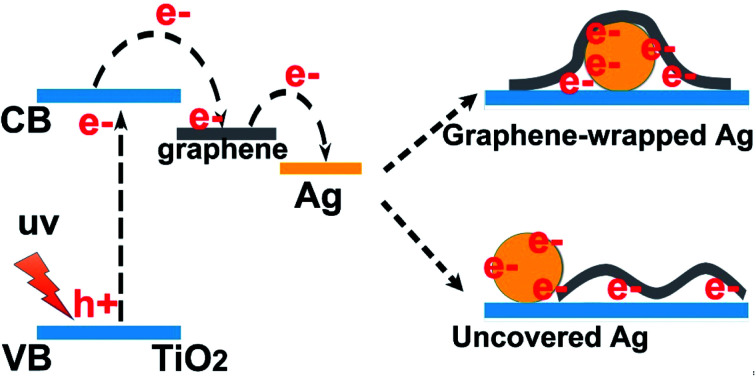

## Introduction

In recent years, graphene-based sheets have been widely used as cocatalysts to improve the photocatalytic properties of semiconductors due to their unique physicochemical properties.^1–4^ To expand their application in photocatalysis areas, graphene-containing binary cocatalysts, in particular those combined with noble metal nanoparticles (such as Au and Ag), have also been adopted to modify the semiconductor photocatalysts such as ZnO, TiO_2_, Bi_2_WO_6_, La_2_Ti_2_O_7_, *etc.*^5–22^ Owing to good electron collection/transport abilities and light response properties of both graphene sheets and noble nanoparticles, these multi-cocatalysts exhibit combined or synergistic effects on improving the performance of these semiconductors in many photocatalytic reactions.

**Fig. 1:**
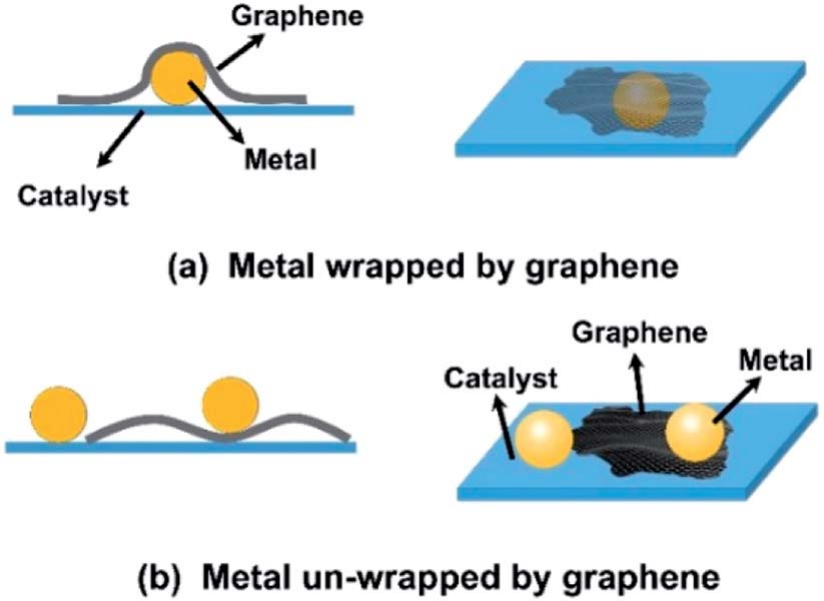
Schematic of the two typical positions of graphene and metal nanoparticles on the surface of photocatalysts: metal nanoparticles (a) wrapped and (b) not wrapped by graphene sheets.

Generally, there are three strategies to obtain graphene and metal-containing composite photocatalysts: (1) using graphene sheets to wrap the metal-loaded semiconductors,^5–9^ (2) using metal nanoparticles to modify graphene–semiconductor composites,^10–17,23,24^ and (3) using a one-step synthesis process, for example, a solvothermal method, to prepare these ternary composites.^25–30^ Although all these methods can produce graphene/metal-containing photocatalysts, the relative positions of these two kinds of cocatalysts on the surface of semiconductors may be slightly different. Since the size of graphene sheets is usually larger than that of metal nanoparticles, there are two typical position states for these two cocatalysts on the surface of the as-modified photocatalysts, in which graphene sheets may or may not wrap the metal nanoparticles, as shown in Fig. 1.

It has been demonstrated that graphene sheets usually function as protecting layers or barriers to hinder the interactions between substances due to their inherent inert characteristics.^31–37^ For example, enwrapping a bacteria in graphene-based sheets could protect the bacteria from outside distractions. Analogously, it could be speculated that in the graphene and metal binary cocatalyst system, when wrapped by graphene sheets (Fig. 1(a)), the noble metal nanoparticles could be protected by these sheets. On the other hand, it is noted that the work functions of graphene and noble metal nanoparticles are different; this can make the electrons to be readily trapped by these noble metal nanoparticles.^10,11^ Thus, the photo-generated electrons that gathered in noble metal nanoparticles could be restricted by graphene sheets to contact the reactants; this might influence the chemical reactions on the surface of these metals, especially those driven by these energetic electrons. However, to date, less attention has been paid to the potential influence of these structural differences of graphene/metal cocatalysts on property improvement of photocatalysts.

Herein, graphene sheets and Ag nanoparticles were used as cocatalysts to modify TiO_2_ spheres, and the photocatalytic reduction of nitro-aromatics to amino-aromatics was chosen as the model reaction. Moreover, two typical composite structures were prepared by adjusting the loading sequence of graphene and Ag nanoparticles on the surface of TiO_2_. It was found that when Ag nanoparticles were loaded first, some of these metal nanoparticles were covered by the subsequently loaded graphene sheets. On the other hand, when the loading order was reversed, most of the Ag nanoparticles were not covered by graphene sheets. Due to wrapping by graphene sheets, the function of Ag in the reaction system was influenced, and the photocatalytic reduction properties of these composites were inferior to those in the case when graphene sheets were loaded first followed by Ag nanoparticles, with the same dosage of the two cocatalysts. Moreover, this barrier effect caused by graphene exists in the typical control reaction using Ag as the main catalyst. Thus, elimination of the possible barrier effects of graphene on the other cocatalyst would be a promising way to further improve the photocatalytic properties of the as-obtained composite, which can be extended to other graphene-involved multi-component catalysis systems in the future.

## Experimental

### Materials

Graphite flakes (325 mesh) were purchased from Alfa Aesar Chemical Corporation. Tetrabutyl titanate (98%), silver nitrate (99.8%), formic acid (98%), and sodium borohydride (96%) were purchased from Shanghai Chemical Corporation. (3-Aminopropyl)-trimethoxysilane (97%) and 4-nitrophenol (99.5%) were purchased from Aladdin Chemical Corporation. All chemicals were used without further treatment.

### Preparation of graphene oxide and TiO_2_ spheres

Graphene oxide (GO) sheets were prepared from natural graphite using Hummers method,^38^ and small GO sheets (about 300–400 nm) were obtained through sonication and centrifugation.^39,40^ TiO_2_ spheres were synthesized using the method reported by Yin and co-authors^41,42^ and then modified by (3-aminopropyl)-trimethoxysilane (APTMS). The details about the preparation of small GO sheets and modification of TiO_2_ can be found in our previous study.^36^

### Preparation of graphene or Ag-containing TiO_2_ binary composites

Graphene-wrapped TiO_2_ samples (labeled as TG) were also prepared according to our previous studies. Typically, a GO dispersion (1.5 mL, 0.33 mg mL^−1^) was added to a TiO_2_ suspension (50 mg of modified TiO_2_ was dispersed in 50 mL of water), which was stirred for 12 h at room temperature to form GO-wrapped TiO_2_ composites (labeled as TGO_0.5_). After this, TGO_0.5_ samples that were dried at 60 °C were redispersed in ethanol (50 mL) and then irradiated with ultraviolet light (16 W, Philips TUV-4W with a wavelength of around 254 nm) for 2 h under a N_2_ atmosphere to obtain TG_0.5_ composites.

Ag-modified TiO_2_ composites were prepared using a modified photo-deposition method. For example, a AgNO_3_ solution (1.0 mL, 0.78 mg mL^−1^) was added to a TiO_2_ suspension (100 mg of modified TiO_2_ was dispersed in 100 mL of ethanol), and the mixed liquid was irradiated with the same ultraviolet light for 2 h under a N_2_ atmosphere. The as-formed Ag-decorated TiO_2_ samples were labeled as TA_0.5._ The subscript in these abbreviations was the added content of GO or/and Ag, and the other graphene or Ag-containing composites with different contents of graphene or Ag were prepared through the same process.

### Preparation of graphene/Ag-containing TiO_2_ ternary composites

The as-obtained binary samples were used as starting materials to prepare our ternary composites. The preparation process of graphene-wrapped TA composites was similar to that of TG, but the pure TiO_2_ spheres were replaced by TA samples, and the final composites were labeled as TA_*x*_G_*y*_ (*x* and *y* wt% were the added contents of Ag and GO, respectively). Similarly, when Ag-modified TG samples were constructed, TGO composites were used as precursors for loading Ag nanoparticles. The photo-deposition process of Ag was consistent with that of TA samples, and the as-prepared ternary samples were labeled as TG_*x*_A_*y*_.

### Photocatalytic reduction of 4-nitrophenol

In a typical reaction, TiO_2_-based samples (*ca.* 40 mg) and formic acid (30 μL) were added to a 4-nitrophenol solution (80 mL, 12 ppm) in a quartz vial. Then, the suspensions were stirred in dark for 0.5 h under N_2_ protection. Ultraviolet light (16 W, Philips TL-4W with a wavelength of around 365 nm) was used as the irradiation source. At a given time interval of light irradiation, the reaction solution was withdrawn and filtered, and the filtrate with the products was analyzed by high-performance liquid chromatography (Waters-2998).

### Reduction of 4-nitrophenol by NaBH_4_

Control tests were carried out according to our previous study.^43^ Typically, aqueous solutions of 4-nitrophenol (0.21 mL, 100 ppm) and NaBH_4_ (0.2 mL, 3.8 mg mL^−1^) were added to deionized water (2.5 mL) in a quartz cuvette. Then, TiO_2_ with different contents of graphene and Ag (0.36 mg dispersed in 0.18 mL of water) was added. After proceeding the reaction for a certain time, the solution was filtered, and the filtrate was analyzed by UV-vis spectroscopy (Thermo Fisher scientific, Genesys 10S).

### Characterization

X-ray diffraction (XRD) measurements were performed using a diffractometer (Bruker D8 Advance) with Cu Kα radiation. Thermogravimetric tests were performed using a thermogravimetric analyzer (Mettler Toledo) from 25 to 800 °C at the heating rate of 5 °C min^−1^ in an air flow. Inductively coupled plasma spectroscopy (ICP, Thermo Fisher Scientific Xseries 2) was used to investigate the content of the metal component. The zeta-potentials of samples were tested by the Malvern Zetasizer Nano-ZS90 particle analyzer. Atomic force microscopy (AFM, Dimension Icon Bruker) in tapping mode was used to measure the size of the GO flakes. X-ray photoelectron spectroscopy (XPS) studies were carried out *via* a Thermo Scientific Escalab 250 X-ray photoelectron spectrometer using Mg Kα (*hν* = 1253.6 eV) X-ray as the excitation source. Raman spectra were obtained from 200 to 2000 cm^−1^*via* a Raman microprobe (Renishaw Invia) using a 514.5 nm argon ion laser. The morphology of composites was analyzed by a transmission electron microscope (TEM, Tecnai G2 F20 S-TWIN) and a field emission scanning electron microscope (FESEM, Hitachi S8010). Photo-electrochemical measurements were carried out using the Autolab electrochemical workstation in a three-electrode cell with 0.2 M Na_2_SO_4_ as the electrolyte. Ag/AgCl in saturated KCl and Pt wire were used as the reference and counter electrodes, respectively. Indium-tin oxide glasses coated with samples were used as the working electrodes. UV light was generated by a 500 W xenon lamp (Beijin Perfectlight, CHF-XM500), and a 0.5 M KCl solution containing 0.01 M K_3_[Fe(CN)_6_]/K_4_[Fe(CN)_6_] was utilized to investigate the Nyquist impedance.

## Results and discussion

As is known, there are many methods to prepare graphene and metal-containing ternary composite photocatalysts. However, less attention is paid to the potential structural difference (derived from the loading strategy) of these two kinds of cocatalysts in composites. In this study, we loaded graphene and metal nanoparticles on semiconductors by a stepwise method. The process for loading each cocatalyst was almost the same, but the loading sequence was reversed. Moreover, to enable graphene sheets to better wrap the metal nanoparticles on the surface of semiconductors, small GO sheets (less than 400 nm, as shown in the AFM images in Fig. S1, ESI†) and TiO_2_ spheres (400 nm) were chosen as the starting materials to construct ternary composites based on our previous research.^36^ In our composite systems, Ag nanoparticles were decorated on the surface of TiO_2_ spheres (or GO-wrapped TiO_2_) by traditional photo-deposition methods in an ethanol solution. On the other hand, these GO sheets (zeta-potential was about −17 mV) that were enwrapping TiO_2_ (+22 mV) or Ag-modified TiO_2_ spheres (+21 mV) through electrostatic adsorption were reduced by a photo-reduction strategy according to previous studies.^44–47^

**Fig. 2:**
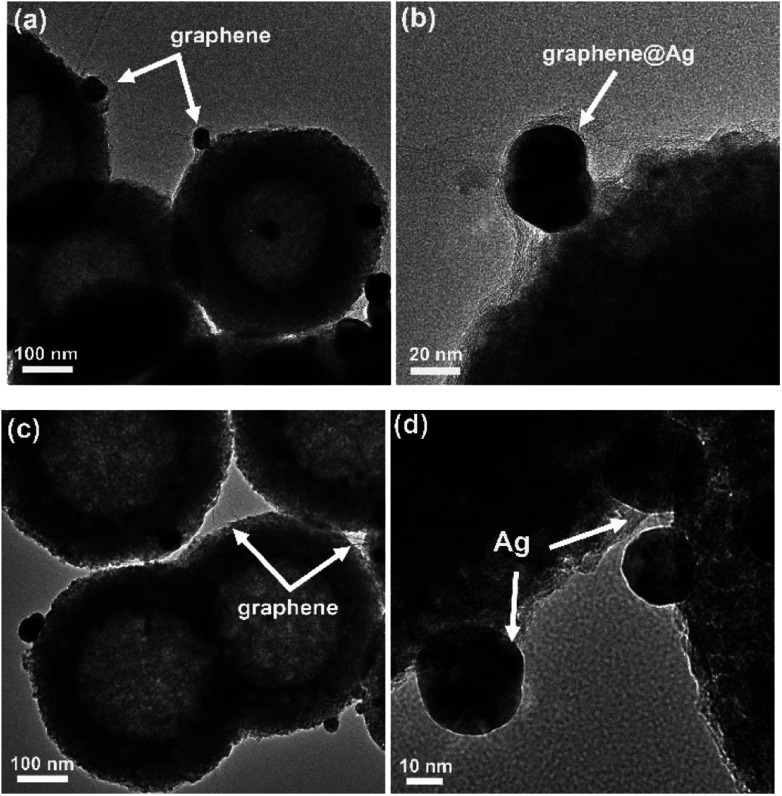
TEM images of the samples. (a) and (b) TA_0.5_G_0.5_, (c) and (d) TG_0.5_A_0.5_.

**Fig. 3:**
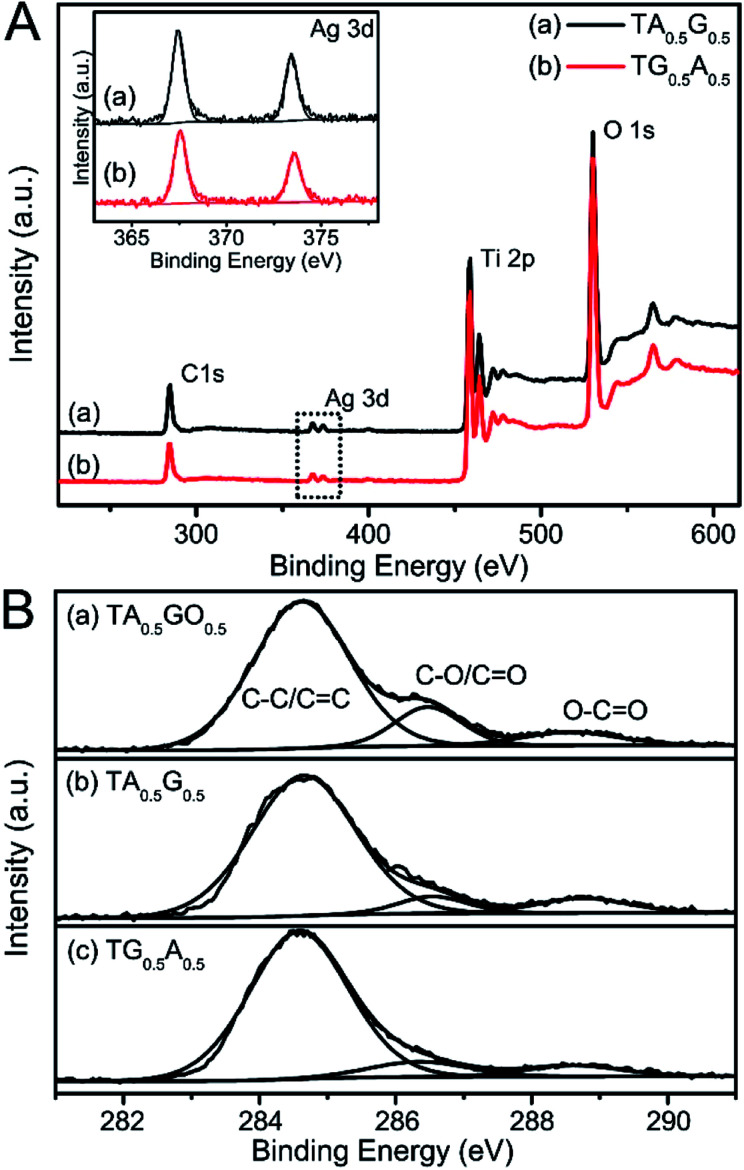
(A) The wide-survey XPS spectra of (a) TA_0.5_G_0.5_ and (b) TG_0.5_ A_0.5_. Inset shows the Ag 3d XPS spectra of samples. (B) C1 XPS spectra of (a) TA_0.5_GO_0.5_, (b) TA_0.5_G_0.5_, and (c) TG_0.5_ A_0.5_.

Fig. 2 displays the TEM images of two typical ternary composites obtained by different loading sequences of GO and Ag nanoparticles on TiO_2_ spheres (the added dosages of both GO and Ag are 0.5 wt%, and XRD patterns of the samples are shown in Fig. S2†). From these images, it was found that both graphene and Ag particles existed on the surface of TiO_2_ spheres (which could also be supported by the wide-survey XPS spectra, Fig. 3A), but with tiny structural differences. In particular, when ternary composites were obtained by pasting graphene sheets on Ag–TiO_2_ composites (TA_0.5_G_0.5_), some Ag nanoparticles on the surface of TiO_2_ were covered by these subsequently loaded graphene sheets, as shown with arrows in Fig. 2(a) and (b). Certainly, this enwrapping process of graphene on the surface of Ag-modified TiO_2_ was random; thus, not every Ag nanoparticle was covered by these carbon sheets. By contrast, since the graphene sheets were already attached on the surface of the TiO_2_ spheres, these subsequently loaded Ag nanoparticles were inclined to load on the surface of the composite spheres. As shown in Fig. 2(c) and (d), almost all the Ag nanoparticles were exposed in TG_0.5_A_0.5_ composites, which were not covered by the already loaded graphene sheets. In theory, it is possible that Ag nanoparticles can decorate the surface of pasted graphene due to its electron collection ability. However, these subsequently loaded Ag nanoparticles could still be considered as unwrapped (Fig. 1(b)).

Although the loading order of graphene and Ag was reversed, their characteristics in the final composites were almost the same (Fig. 2, 3, S2 and S3†). For example, from TEM images, we can find that the sizes of the photo-deposited Ag nanoparticles in both TG_0.5_A_0.5_ and TA_0.5_G_0.5_ samples are similar to each other, which are about 30 nm. Moreover, the inductively coupled plasma analysis suggested a similar content of Ag in these two composites. To investigate the changes in GO sheets during the preparation process, we also carried out XPS and Raman analyses. Fig. 3B shows the C1s XPS spectra of the typical TG_0.5_A_0.5_ and TA_0.5_G_0.5_ samples, and TA_0.5_GO_0.5_ is used as the control sample. As is known, after a certain degree of reduction, the areas of oxygen-containing functional groups decrease in the C1s spectra.^48,49^ By comparing the changes in these groups (C–O/C=O) in the TA_0.5_GO_0.5_ and TA_0.5_G_0.5_ samples, it was found that these added GO sheets could be reduced through photocatalytic reduction (Fig. 3B(a) and (b)), which was consistent with previous studies.^44,45^ Moreover, similar C1s spectra in both TA_0.5_G_0.5_ and TG_0.5_A_0.5_ indicated that GO sheets in these two kinds of ternary composites possessed approximately the same reduction degree, which could be further supported by Raman results (Fig. S3†).^50,51^ In addition, the contents of graphene sheets in TA_0.5_G_0.5_ and TG_0.5_A_0.5_ composites were similar to each other, which were confirmed by thermogravimetric analysis (Fig. S4†).

It was shown that the characteristics of each kind of cocatalyst were similar; however, the structures, especially the relative positions, of graphene and Ag were indeed different in these two kinds of ternary composites (as suggested by TEM images in Fig. 2). Unfortunately, in most composites, these subtle differences in structures have not attracted enough attention, and their influence on the photocatalysis performance has not been considered.

**Fig. 4:**
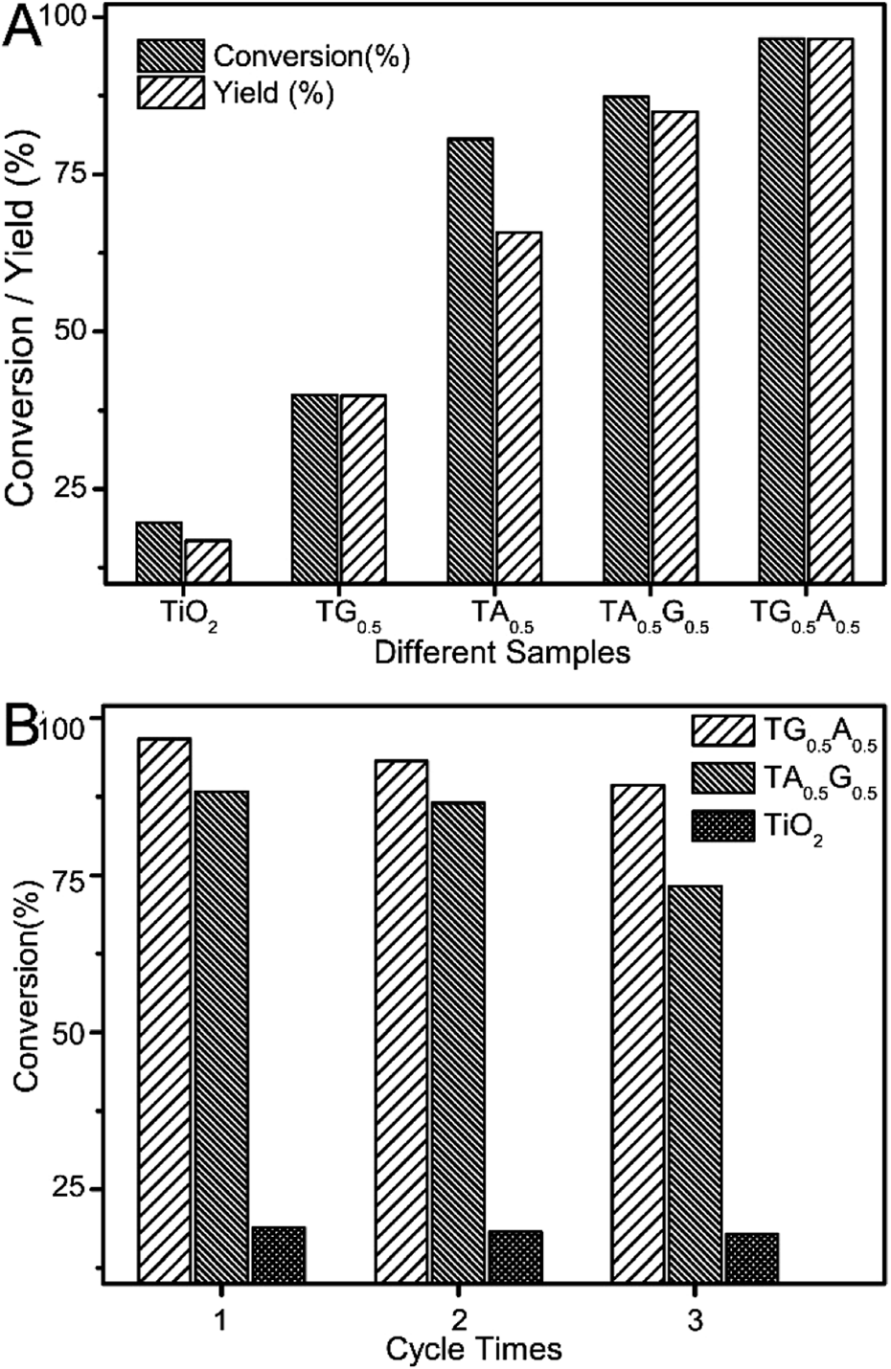
(A) Photocatalytic conversion of 4-nitrophenol to 4-aminophenol using some typical composites. The content of GO and/or Ag was about 0.5 wt% in all the composite photocatalysts. (B) Conversion rate of recycle experiments using TG_0.5_A_0.5_, TA_0.5_G_0.5_ and TiO_2_ as photocatalysts.

**Fig. 5:**
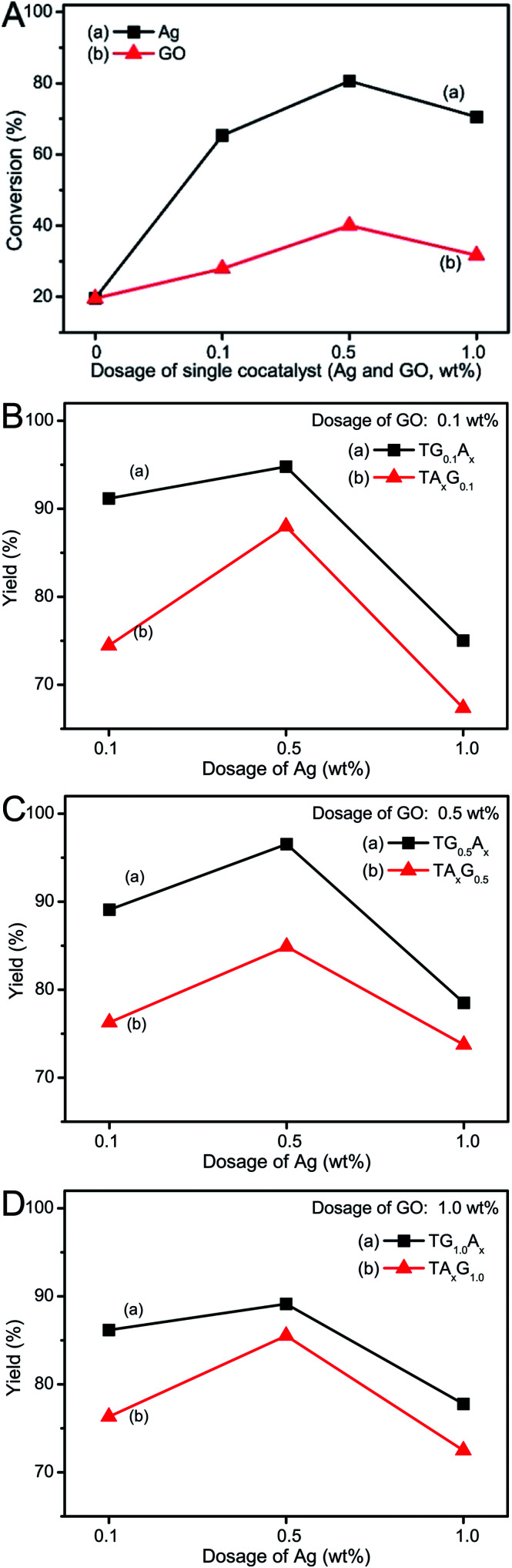
Photocatalytic conversion of 4-nitrophenol using different composites. (A) TiO_2_ with a single cocatalyst (a) Ag and (b) graphene; (B–D) the dosage of GO sheets in each figure was fixed, which was 0.1, 0.5 and 1.0 wt% in (B), (C), and (D) respectively. However, the dosage of Ag was adjusted from 0.1 to 1.0 wt% in the two kinds of ternary composites: (a) TGA and (b) TAG samples.

As demonstrated in many studies, either graphene sheets or noble metals can improve the photocatalytic reduction performance of semiconductors for reducing nitro-aromatics to corresponding amino-aromatics with the assistance of a hole scavenger; this is mainly due to their excellent photo-generated charge separation/transport abilities.^36,52,53^ We also carried out similar tests using graphene or Ag-modified TiO_2_ samples as control photocatalysts. It was found that when the content of GO or Ag was about 0.5 wt% in our system, TG_0.5_ or TA_0.5_ composites possessed optimal photocatalytic reduction properties as compared to pure TiO_2_ (Fig. 5A). Certainly, when graphene and Ag were utilized together to modify TiO_2_, the as-obtained ternary composites possessed higher photocatalytic reduction abilities as compared to TiO_2_ with a single cocatalyst; this was probably due to the combined effects of these two cocatalysts. Fig. 4A summarizes the photocatalytic performances of some typical composites towards the reduction of 4-nitrophenol to 4-aminophenol. It could be clearly seen that the photocatalytic performance of TA_0.5_G_0.5_ or TG_0.5_A_0.5_ samples with two cocatalysts was higher than that with a single cocatalyst (TA_0.5_ and TG_0.5_).

It is worth noting that there are some differences between the catalytic activities of TA_0.5_G_0.5_ and TG_0.5_A_0.5_ composites. By comparison, the conversion of 4-nitrophenol using TG_0.5_A_0.5_ composite was *ca.* 97% after irradiation for 9 min, which was about 10 points higher than that of TA_0.5_G_0.5_. Fig. 4B shows the recycle experiment results of the two typical ternary composites. The photocatalytic activities of each composite decreased slightly after every recycle experiment; this was probably due to mass loss of the catalysts. However, the photocatalytic performance of TG_0.5_A_0.5_ was always higher than that of the TA_0.5_G_0.5_ samples after each cycle (the corresponding yield of 4-aminophenol is listed in Fig. S5†).

Furthermore, we investigated the photocatalytic reduction properties of other ternary composites with different contents of graphene and Ag nanoparticles, and the corresponding results are summarized in Fig. 5 (B–D). As expected, these results still supported the fact that when the dosages of each cocatalysts were the same, the photoactivity of Ag-modified graphene-wrapped TiO_2_ spheres (TG_*y*_A_*x*_) was always higher than that of the graphene-wrapped Ag–TiO_2_ composites (TA_*x*_G_*y*_). Taking the dosage of GO precursor as about 0.5 wt% for example, when the dosage of Ag was changed from 0.1 to 1.0 wt%, the photocatalytic performance of TG_0.5_A_*x*_ was higher than that of TA_*x*_G_0.5_ (Fig. 5C). Certainly, an analogous tendency was found in other composites when the dosage of GO was adjusted to 0.1 or 1.0 wt% (Fig. 5B, D and S6†).

**Fig. 6:**
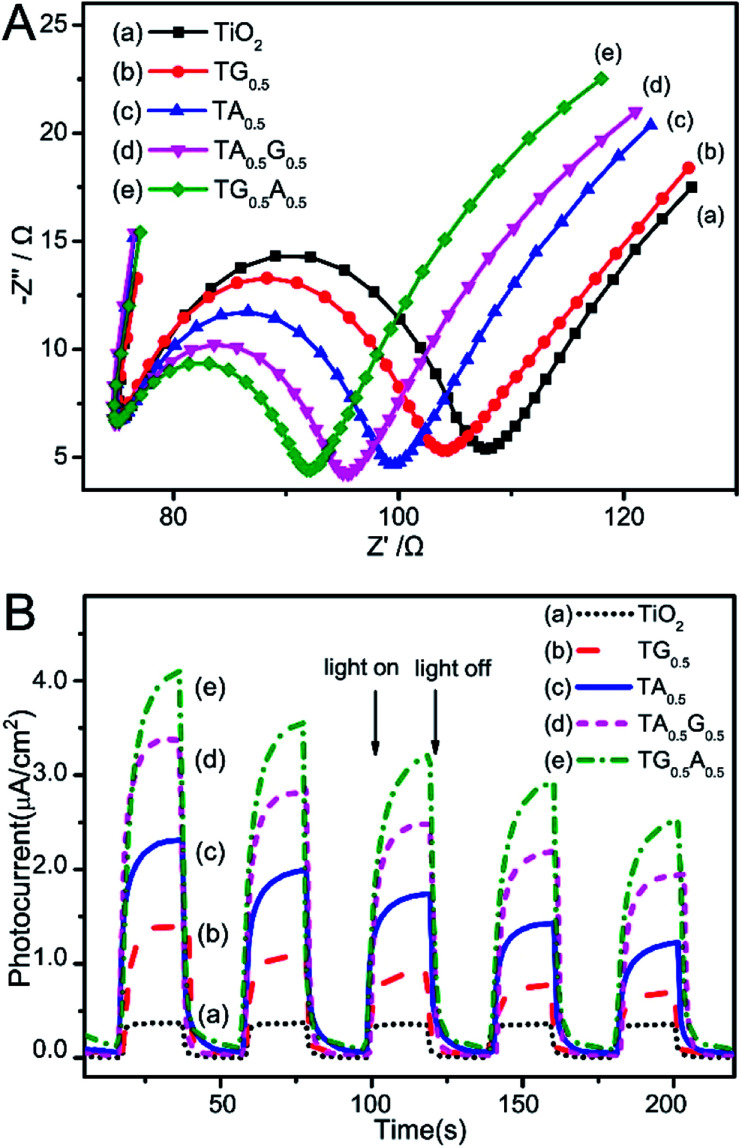
(A) Nyquist impedance plots and (B) photocurrent signals of the samples. (a) TiO_2_, (b) TG_0.5_, (c) TA_0.5_, (d) TA_0.5_G_0.5_, and (e) TG_0.5_A_0.5_.

**Fig. 7:**
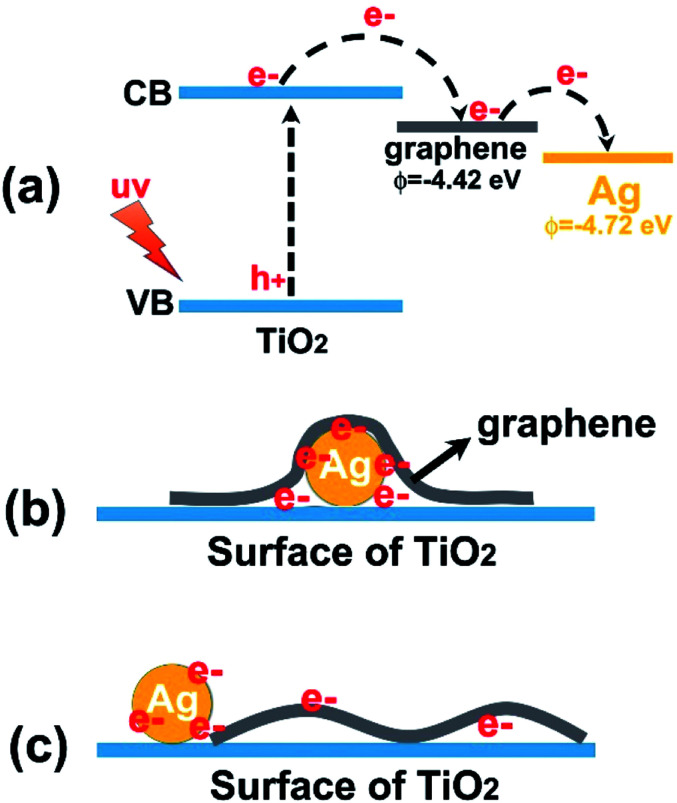
Schematic showing the proposed mechanisms for (a) the transfer of electrons from different energy levels of the ternary heterostructure, and the proposed states of electrons in the two cocatalysts in (b) TAG and (c) TGA samples.

We attribute these differences to the possible barrier effect of graphene sheets on these Ag nanoparticles that might hinder the effective contact of Ag with external environment. As demonstrated recently, graphene-based sheets can function as barrier materials owing to their inherent structure and properties.^31–37,54^ Thus, it can be speculated that when graphene sheets encapsulate Ag nanoparticles on the surface of TiO_2_, certain surface performances of these Ag particles can be disturbed by these barrier-like carbon sheets. Fig. 6A shows the results of electrochemical impedance spectroscopy (EIS) experiments of our samples, which demonstrates the charge-carrier migration process at the contact interface between the electrode and electrolyte solution. By comparing the Nyquist plots, especially the signals of the two ternary composites, it was found that the TG_0.5_A_0.5_ composite possessed more depressed semicircles than TA_0.5_G_0.5_; this indicated that faster interfacial electron transfer was obtained over TG_0.5_A_0.5_ than that over TA_0.5_G_0.5_; this was probably due to the undisturbed charge transport between the uncovered Ag nanoparticles and electrolyte in the TG_0.5_A_0.5_ samples. On the other hand, as is known, both graphene and Ag can collect photo-generated electrons and promote reactions on their surfaces in photocatalysis systems. However, when graphene sheets are in contact with Ag nanoparticles, photo-electrons are inclined to be trapped by Ag due to the work function difference between graphene (−4.42 eV, *vs.* vacuum) and Ag (−4.72 eV),^10,11^ as shown in the scheme in Fig. 7(a). Thus, the transport and utilization of photo-generated electrons that gather in these Ag nanoparticles could be restrained in these TAG composites.^55^ From the photocurrent results shown in Fig. 6B, it was found that the photocurrent intensity of TA_0.5_G_0.5_ was indeed lower than that of TG_0.5_A_0.5_ samples in our system. Accordingly, the photocatalytic reactions driven by these photo-generated electrons were also disturbed in the TAG samples; this resulted in a decrease in the catalytic performance as compared to that of the TGA samples.

**Fig. 8:**
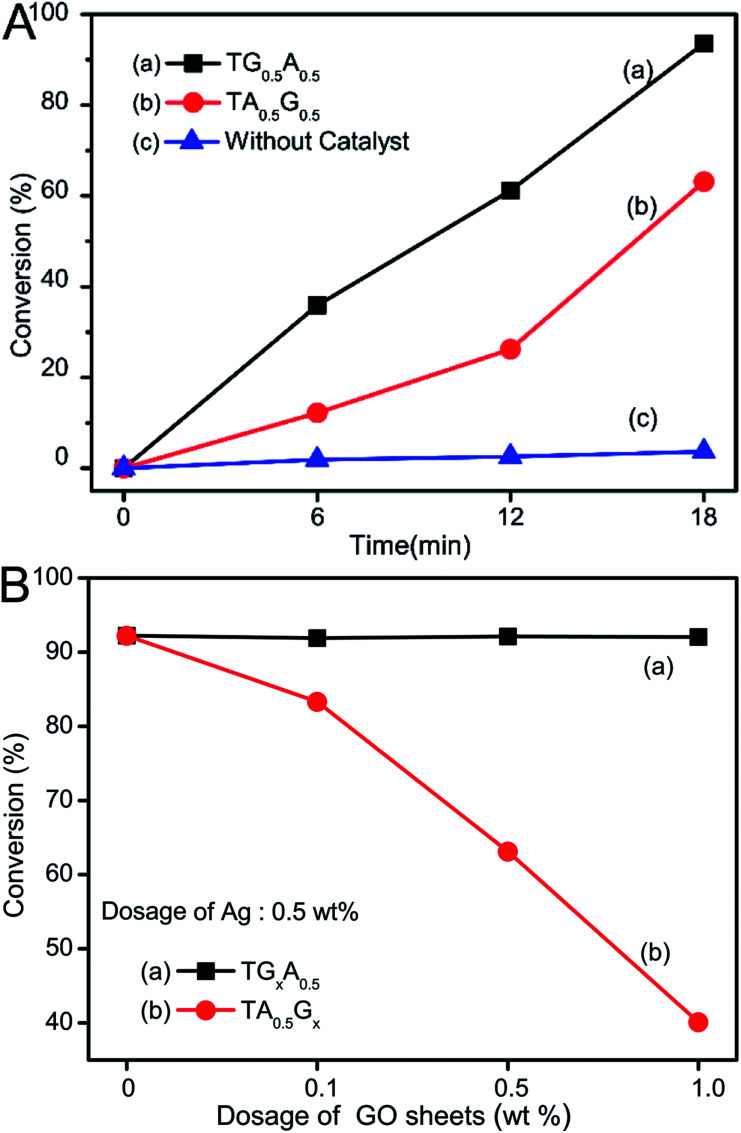
(A) Conversion rate of 4-nitrophenol as a function of time after the addition of NaBH_4_: (a) TG_0.5_A_0.5_, (b) TA_0.5_G_0.5_, and (c) control test without a catalyst. (B) Conversion rate of 4-nitrophenol after reaction for 18 min using our samples with different dosages of GO sheets: (a) TG_*x*_A_0.5_ and (b) TA_0.5_G_*x*_ (*x* = 0, 0.1, 0.5, and 1.0 wt%).

Furthermore, the barrier effect of graphene sheets on these Ag nanoparticles could be further confirmed by a benchmark reduction of 4-nitrophenol to 4-aminophenol using sodium borohydride as the reductant.^43,56–58^ As is well-known, this reaction is rapid in the presence of metallic surfaces. Thus, it can be speculated that when their surfaces are enwrapped (Fig. 1), the performance of Ag can be restrained. Taking the 0.5 wt% dosage of both graphene and Ag for example, TG_0.5_A_0.5_ samples could convert 92% of 4-nitrophenol after reacting for 18 min (Fig. 8A(a)), whereas only about 60% of 4-nitrophenol was converted when TA_0.5_G_0.5_ was used as a catalyst (Fig. 8A(b)). Moreover, since the Ag nanoparticles are not wrapped by graphene sheets, the catalytic activity of Ag nanoparticles in TG_*x*_A_0.5_ was hardly disturbed by the dosage change of GO sheets (Fig. 8B(a)). However, the catalytic properties of TA_0.5_G_*x*_ composites decreased obviously with an increase in the dosage of GO sheets (Fig. 8B(b)); this was because more Ag particles on the surface of TiO_2_ were wrapped by these graphene sheets.

Accordingly, our study has shown that in a graphene/Ag-containing photocatalysis system, the enwrapping of graphene sheets on Ag nanoparticles could be controlled by simply adjusting the loading order of these two kinds of cocatalysts on the surface of TiO_2_ semiconductors (Fig. 7(b) and (c)). Since Ag nanoparticles are not wrapped by graphene sheets, the features of Ag as cocatalysts could be developed efficiently; this further improved the photocatalytic performance of these as-obtained ternary composites. While constructing composites containing multi-cocatalysts in the future, the possible negative effects of graphene sheets on other cocatalysts are worth taking into account; this would be helpful to better utilize the functions of the added cocatalysts and improve the properties of composites.

## Conclusion

In this study, we have prepared graphene and Ag-containing TiO_2_ composite photocatalysts with tiny structural differences by stepwise photoinduced loading methods. When Ag was loaded on TiO_2_ first, the subsequently added graphene-based sheets could wrap some Ag nanoparticles. However, when the loading order was reversed, the Ag nanoparticles were not covered by these pre-coated carbon sheets. Since graphene sheets usually function as barrier-like layers, when they wrap some Ag nanoparticles in TAG samples, the performance of these Ag particles as cocatalysts is affected, especially the reactions occurring on their surfaces. Based on the results of photocatalytic reduction of 4-nitrophenol, it was shown that these TGA samples with uncovered Ag nanoparticles possessed better photocatalytic properties than the graphene-wrapped TAG composites in the case of the same dosage of these two cocatalysts. Moreover, the barrier effect of graphene in our samples could be found in the typical chemical reduction of 4-nitrophenol using Ag as the catalyst.

Our study demonstrated that in a composite system with graphene-containing multi cocatalysts, by avoiding the potential influence of graphene on other cocatalysts, the properties of composites could be further improved, which provided us a new strategy to construct efficient composite materials in the future.

## Conflicts of interest

There is no conflict of interest.

## Supplementary Material

RA-008-C8RA02268B-s001
