# Supplementary material for: Reducing the barrier effect of graphene sheets on a Ag cocatalyst to further improve the photocatalytic performance of TiO2
Source: RSC Adv. 2018 Apr 16;8(25):14056–63. doi: 10.1039/c8ra02268b (PMC9079882; doi:10.1039/c8ra02268b)
Supplement: RA-008-C8RA02268B-s001 [file RA-008-C8RA02268B-s001.pdf]

Electronic Supplementary Material (ESI) for RSC Advances.
This journal is © The Royal Society of Chemistry 2018

Reducing the barrier effect of graphene sheets on Ag cocatalyst to further improve the photocatalysis performance of TiO_2

Juanjuan Ma, [a] Chaocun Zhou, [a] Jinlin Long, [a] Zhengxin Ding,[a] Rusheng Yuan,[a] Chao Xu [a]

[a] State Key Laboratory of Photocatalysis on Energy and Environment, College of Chemistry, Fuzhou University, Fuzhou 350002, P. R. China.

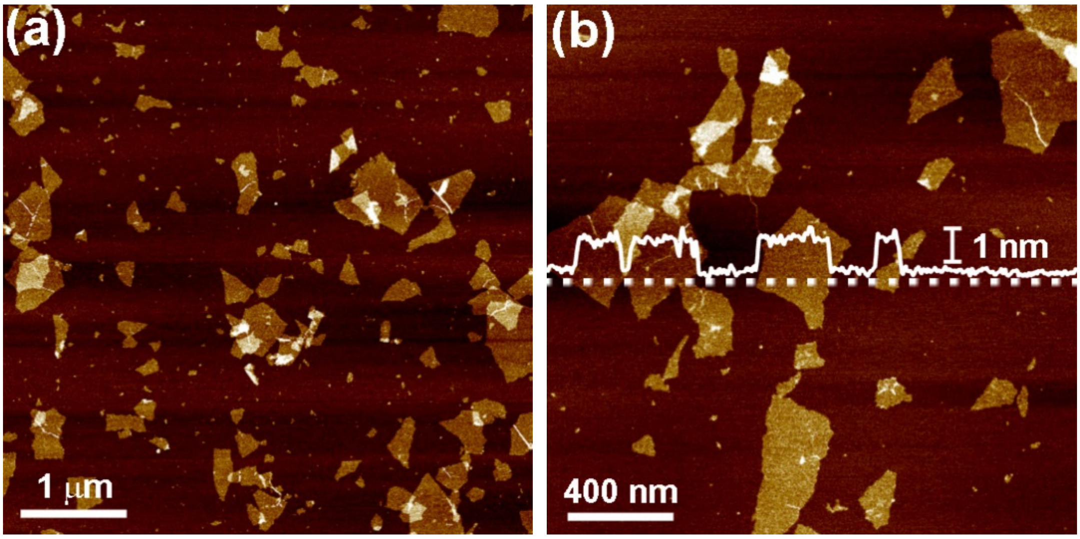

Fig. S1. AFM images of small GO sheets. White curve in Figure (b) is the height profile taken along the dotted line (scale bar, 1 nm). Samples were prepared by drop-casting dilute GO sheet dispersions onto mica plates.

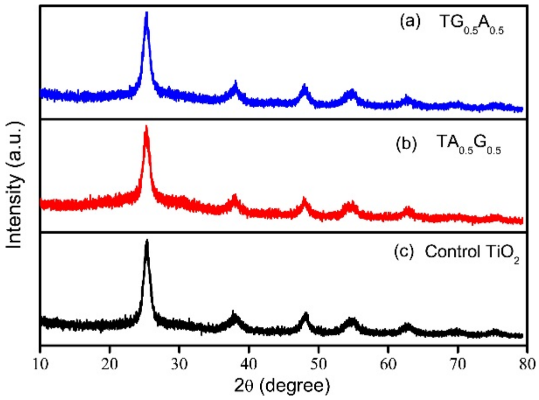

Fig.S2. XRD patterns of samples. (a) $TG_{0.5}A_{0.5}$, (b) $TA_{0.5}G_{0.5}$ and (c) control TiO_2 spheres

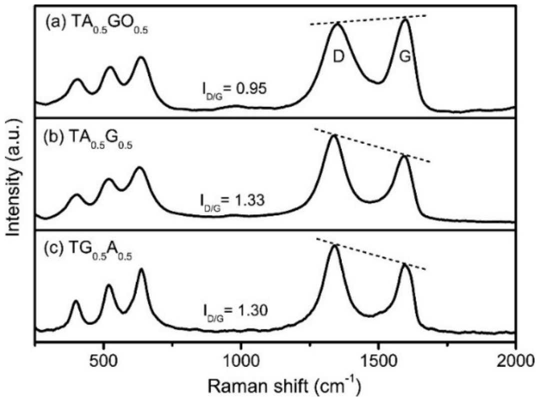

Fig. S3. Raman spectra of samples. (a)$TA_{0.5}GO_{0.5}$, (b) $TA_{0.5}G_{0.5}$ and (c) $TG_{0.5}A_{0.5}$

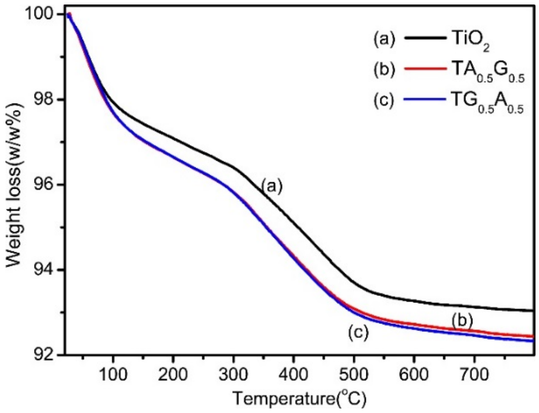

Fig. S4. Thermogravimetric analysis of (a) TiO_2, (b) $TA_{0.5}G_{0.5}$ and (c) $TG_{0.5}A_{0.5}$ samples

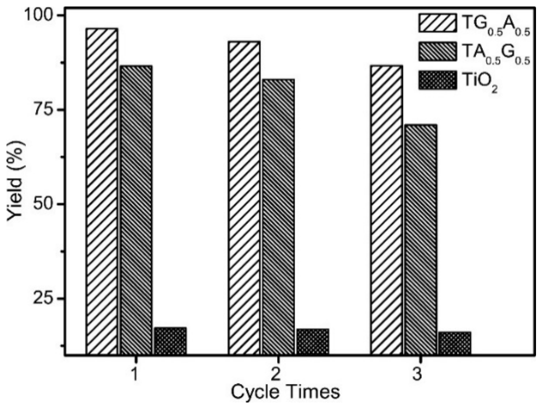

Fig. S5. Yields of 4-aminophenol of recycle experiments using $TG_{0.5}A_{0.5}$, $TA_{0.5}G_{0.5}$ and TiO_2 as photocatalysts.

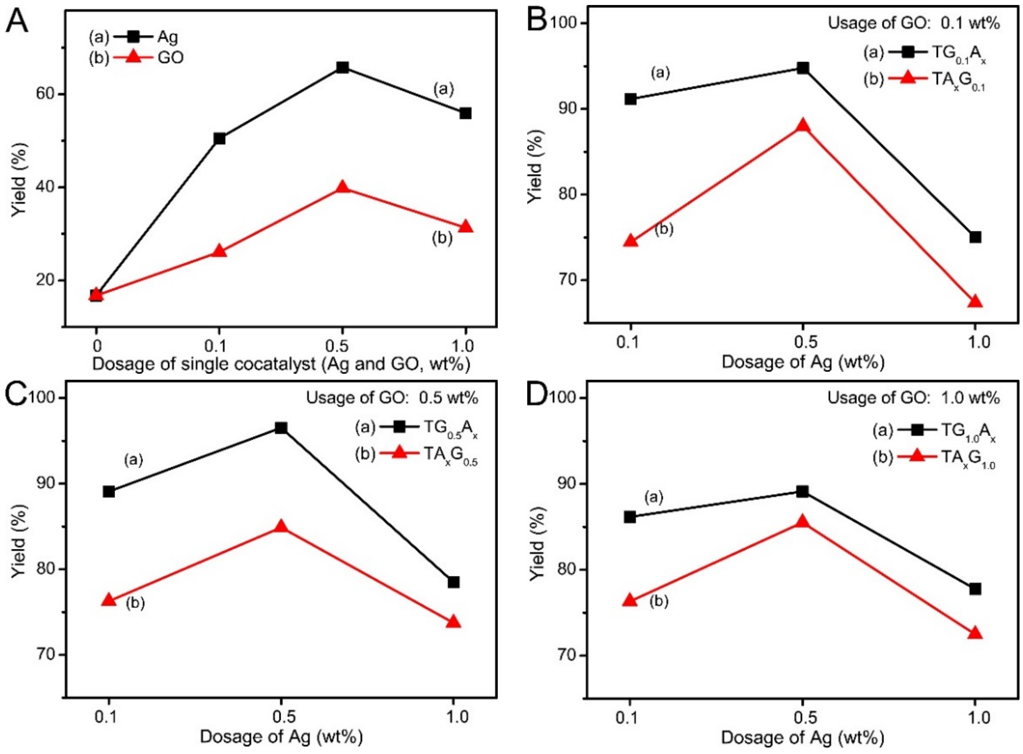

Fig. S6. Yields of 4-aminophenol using different composites. (A) TiO_2 with different dosage of single cocatalyst (a) Ag nanoparticles and (b) graphene; (B-D) The usage of GO sheets in each figure is fixed, which is 0.1, 0.5 and 1.0 wt % in (B), (C) and (D) respectively. While the dosage of Ag is adjusted from 0.1 to 1.0 wt % in two kinds of ternary composites: (a) TGA and (b) TAG samples.
